# Case report: A novel *de novo* deletion mutation of *DYRK1A* is associated with intellectual developmental disorder, autosomal dominant 7

**DOI:** 10.3389/fnins.2023.1174925

**Published:** 2023-05-19

**Authors:** Cong Zhou, Hongmei Zhu, Qinqin Xiang, Jingqun Mai, Xihan Wang, Jing Wang, Shanling Liu

**Affiliations:** ^1^Department of Medical Genetics, Prenatal Diagnostic Center, West China Second University Hospital, Sichuan University, Chengdu, China; ^2^Key Laboratory of Birth Defects and Related Diseases of Women and Children, Ministry of Education, Sichuan University, Chengdu, China

**Keywords:** microcephaly, *DYRK1A*, deletion mutation, whole-exome sequencing, intellectual developmental disorder 7

## Abstract

**Background:**

Intellectual developmental disorder 7 (also named *DYRK1A* syndrome) is an autosomal dominant disease. The main clinical features of *DYRK1A* syndrome include intellectual disability, microcephaly, and developmental delay. This study aimed to identify pathogenic variants in a Chinese girl with developmental delay, impaired social interaction, and autistic behavior.

**Case presentation:**

The case was a 6-year-old girl. Clinical symptoms of the patient mainly included developmental delay, seizures, autistic behavior and impaired social interaction. The patient presented with microcephaly, bushy eyebrows, a short lingual frenum, binocular esotropia, bilateral valgus and external rotation, and walked with an abnormal gait. Using whole-exome sequencing, we identified a 9,424 bp *de novo* heterozygous deletion (containing coding exons 10, 11, and 12, and partial sequences of non-coding exon 12) in *DYRK1A*, which is responsible for *DYRK1A* syndrome. The *DYRK1A* variant is classified as pathogenic according to the criteria of the American College of Medical Genetics and Genomics.

**Conclusions:**

The findings of this study augment the data regarding the pathogenic variants of *DYRK1A* and provide important information for molecular diagnosis.

## 1. Introduction

Intellectual developmental disorder 7 (*DYRK1A* syndrome; OMIM: 614104) is an autosomal dominant disorder, which is characterized by primary microcephaly, developmental delay, intellectual disability, delayed language development, seizures, and autistic behavior (Arbones et al., 2019). *DYRK1A* syndrome is caused by heterozygous variation in *DYRK1A* (dual-specificity tyrosine phosphorylation-regulated kinase 1a; OMIM: 600855) (van Bon et al., 2011). *DYRK1A* has been mapped to chromosome 21q22.13; it contains 12 exons and codes for the DYRK1A protein containing 763 amino acids (NM_001396.5) (Shindoh et al., 1996; Guimera et al., 1999). This protein is highly conserved and plays an essential role in the development of the central nervous system (van Bon et al., 2011; Arbones et al., 2019). DYRK1A is a regulator of brain growth and function—including neurogenesis, neuronal proliferation, and differentiation—synaptic transmission, and neurodegeneration (Widowati et al., 2018; Arbones et al., 2019).

*DYRK1A* syndrome is typically caused by a *de novo* pathogenic variant. Evaluations of the genetic basis of *DYRK1A* syndrome have revealed truncating, missense, frameshift, insertion, deletion mutations, and complex rearrangements in *DYRK1A*, all of which result in varying *DYRK1A* syndrome phenotypes. To the best of our knowledge, partial *DYRK1A* deletions is very rare. According to the literature included in human gene mutation database (HGMD), only two patients of partial *DYRK1A* deletions have been reported (van Bon et al., 2011; Abe-Hatano et al., 2021).

We report on a patient who presented primary microcephaly, developmental delay, impaired social interaction, and autistic behavior. In the patient, a *de novo* 9,424 bp heterozygous deletion in *DYRK1A* was detected by whole-exome sequencing (WES). The deletion removed coding exons 10 to 12 and partial sequences of non-coding exon 12. The findings of this study augment the data regarding pathogenic variants of *DYRK1A* and provide important information for molecular diagnosis.

## 2. Patients and methods

### 2.1. Patients

A Chinese adult female and her child sought genetic counseling from the Department of Medical Genetics of West China Second University Hospital of Sichuan University (Chengdu, China). The child was a 6-year-old girl diagnosed with developmental delay, impaired social interaction, and autistic behavior. Physical examination showed that she had bushy eyebrows, a short lingual frenum, binocular esotropia, bilateral valgus and external rotation, and walked with an abnormal gait. She was 113 cm in height (<25th percentile), 21.5 kg (normal for her age) in weight, with a body mass index of 16.8 kg/m^2^, and a head circumference of 49 cm (≤−3 standard deviations, SD). She showed developmental delay, had difficulty in speaking and making social interactions, and presented autistic behavior.

**Figure 1:**
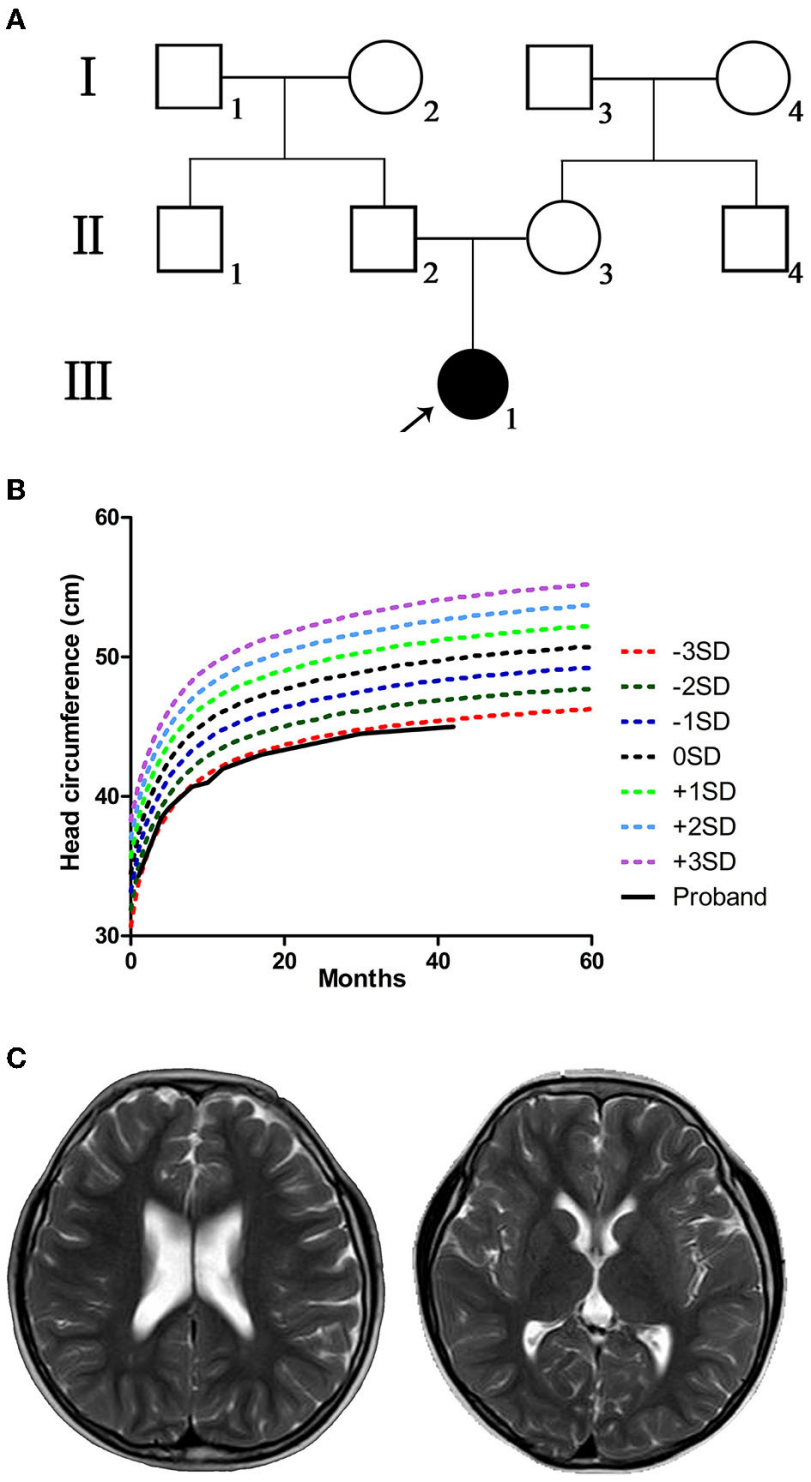
Pedigree, head circumference, growth curve, and brain resonance images (MRI) of the proband. **(A)** Pedigree of the proband's family; black-filled shapes represent the proband, and the unfilled shapes represent the unaffected family members. Males are represented by squares, females by circles. **(B)** The proband with small head circumference grew slowly and lagged markedly behind the referent range. **(C)** The MRI showed bilaterally widened lateral ventricles but without structural anomalies when the proband was 6 years old.

The proband was the first-born child of non-consanguineous Chinese parents from Sichuan Province. The parents were healthy and had no family history of congenital malformation or genetic abnormalities (Figure 1A). In the middle and late stages of the mother's pregnancy, fetal ultrasound indicated several times that the biparietal diameter was less than the average. The proband was born by cesarean section at 41 weeks with a weight of 3,150 g, length of 50 cm, and no history of complications.

The proband passed otoacoustic emission bilateral hearing screening at birth. At 1 month of age, she could not follow objects, and her head circumference was smaller (≤−2 SD) than that of children of the same age. The proband presented binocular esotropia at the age of 5 months. The examination at our hospital showed high muscle tension and hyperactivity of tendon reflex. The proband presented microcephaly (≤−3 SD) when she was 8 months old. The head circumference grew slowly and lagged obviously behind the referent range (WHO multicentre growth reference study group, 2006) in later years (Figure 1B). At the age of 9 months, tests suggested backward motor development, low muscle strength of limbs, and binocular hyperopia. At the age of 14 months, she had mild motor retardation and severe language retardation. At 1 and 2 years of age, she regularly suffered febrile seizures lasting for several seconds, presenting sudden stiffness and staring eyes. The electroencephalogram (EEG) readings were normal. Levetiracetam was used to treat epilepsy. The curative effect of the drug was acceptable, and there were no seizure symptoms during the medication period. After the sudden withdrawal of drugs at the age of 5, seizures re-occurred. The main symptoms were sudden falling to the ground or sudden shaking of hands, and crying after falling to the ground. When she was 5 years old, the assessment showed that the patient's intelligence quotient (IQ) was 79 (critical level), with distraction and mild autism; motor, language, hand-eye coordination, visual performance, and practical reasoning fell below the normal range. When she was 6 years old, cerebral magnetic resonance imaging (MRI) showed bilaterally widened lateral ventricles without structural anomalies (Figure 1C). The clinical features and neurological findings of the patient are summarized in Table 1.

**Table 1:** The proband's main symptoms.

**Clinical symptoms**	
Sex	Female
Age (Yr)	6
Height (cm)	113
Weight (kg)	21.5
Head circumference (cm)	49
Microcephaly	+
Bushy eyebrows	+
Short lingual frenum	+
Binocular esotropia	+
Bilateral hyperopia	+
Bilateral valgus and external rotation	+
**Neurological findings**	
Bilateral ventricular enlargement	+
Brain resonance images showed delayed development of brain myelin sheath (5 months old)	+
Intellectual disability	+
Delayed language development	+
Seizures	+
Normal EEG	+
Hypermyotonia	+
Tendon hyperreflexia	+
Low muscle strength	+
Abnormal gait	+
**Behavioral manifestations**	
Autistic	+
Hyperactivity	+

+, presence of feature.

## 3. Methods

### 3.1. Whole-exome sequencing

Genomic DNA (gDNA) was extracted from the peripheral blood of the proband and her parents and subjected to WES in trio. Nano WES Human Exome V1 (Berry Genomics, Beijing, China) was used to capture the targeted gDNA of the family members according to the manufacturer's instructions. The double-end sequencing program was performed on the Illumina NovaSeq6000 platform (Illumina, Inc. Forster City, CA, USA), and sequence reads of 150 bp were received. The reads were mapped to GRCh38/hg18 using the Burrows-Wheeler Aligner software tool (https://www.plob.org/tag/bwa). The Genome Analysis Tool Kit Unified Genotyper (https://www.broadinstitute.org/gatk/) was used to identify the variants, and variants were annotated using Annovar software (http://annovar.openbioinformatics.org/en/latest/). All variants were filtered based on the frequency in the 1,000 Genomes Project, gnomAD, and ExAC. Variants with a minimum allele frequency (MAF) <0.05 were retained. The variants were evaluated based on OMIM, ClinVar, and the Human Gene Mutation (HGMD) databases (https://my.qiagendigitalinsights.com/bbp/view/hgmd/pro/start.php). The American College of Medical Genetics and Genomics (ACMG) guidelines (Richards et al., 2015) were used to interpret sequence variants.

### 3.2. Quantitative polymerase chain reaction

Quantitative polymerase chain reaction (qPCR) was performed using the family members' gDNA to verify the WES results using SYBR Green qPCR Master Mix (Thermo Fisher Scientific, Vilnius, Lithuania) and an Applied Biosystems 7500 Real-Time PCR System (Thermo Fisher Scientific, Waltham, MA, USA). Each sample was tested in triplicate. The 2[-Delta Delta C(T)] analysis method (Livak and Schmittgen, 2001; Rao et al., 2013) was used to evaluate the copy number of *DYRK1A* exons 9, exons 10, 5′ end of exon 12, and 3′ end of exon 12 in each sample using specific primer pairs (Supplementary Table S1).

## 4. Genetic findings

**Figure 2:**
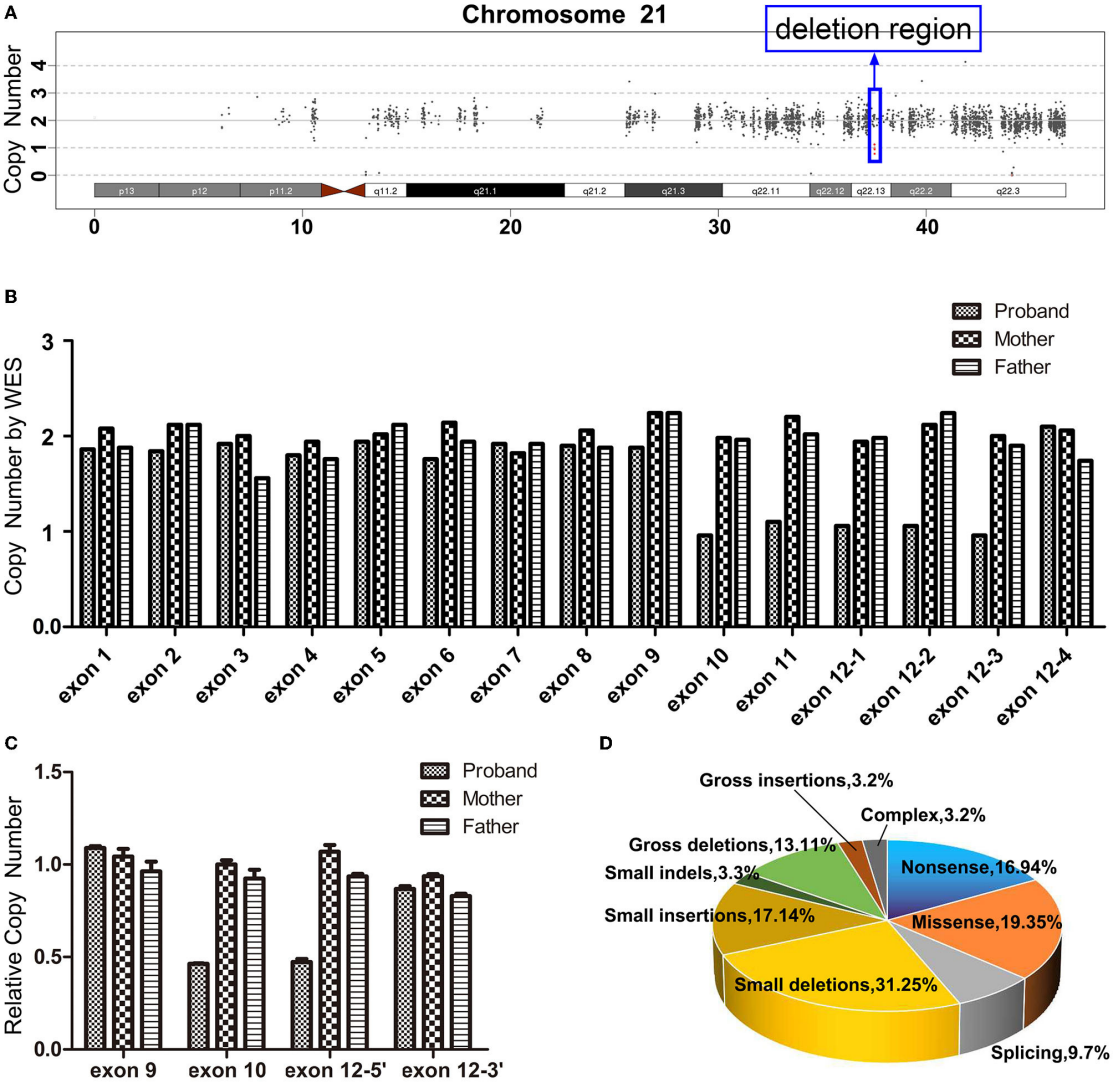
**(A)** Whole-exome sequencing (WES) scatter plot describing the heterozygous loss of 21q22.13 (~9,424 bp) in the proband. **(B)** The exon copy number variations following WES. Exons 1–11 each contain one capture probe; exon 12 contains four probes, exons 12-1(chr21:37511910-37512770), 12-2(chr21:37512771-37513102), 12-3(chr21:37513241-37514706), and 12-4(chr21:37514829-37515376). All the sequences of exon 10 (chr21:37505282-37505589) and exon 11 (chr21:37506098-37506223), and most sequences of exon 12 (chr21:37511910-37514706) in *DYRK1A* presented only a single copy in the proband, and the rest of the *DYRK1A* gene presented exhibited a copy number of two. Both maternal and paternal data revealed normal copy numbers for the whole exon of the gene. **(C)** Heterozygous deletion of the distal part of *DYRK1A* (NM_001396.5) was validated by quantitative polymerase chain reaction. The proband exhibited half the number of copies for exon 10 and 5′ end of exon 12 when compared to her mother and father. However, there is no significant difference in the copy number at exon 9 and the 3 'end of exon 12 compared to her parents. **(D)** The ratio of disease mutation types of *DYRK1A* according to the Human Gene Mutation database.

There is not any single nucleotide variation with pathogenicity or likely pathogenicity in the WES (the summary of the WES on the patient's family members is listed in Supplementary Table S2). However, the WES data produced scatter diagrams which indicated that the proband experienced a variation, with a 9,424 bp heterozygous deletion (hg38: chr21:37,505,282_37,514,706) on chromosome 21q22.13, with a copy number of 1 (Figure 2A). The deletion region contained coding exons 10, 11, and 12, and partial sequences of non-coding exon 12 in *DYRK1A* (NM_001396.5). The copy number of all sequences of exons 1–9 and the partial sequences of exon 12 was two. However, the maternal and paternal data revealed two copies of each exon (Figure 2B). qPCR revealed that the proband presented half the number of copies of exon 10 and 5′ end of exon 12 found in her mother or father (Figure 2C). Taken together, these results indicate that the patient experienced a heterozygous loss of all the sequences of exon 10, the sequences of exon 11, and partial sequences of exon 12 in *DYRK1A*, whereas both her parents presented with a normal genotype.

## 5. Discussion

*DYRK1A* is implicated in the regulation of apoptosis and proliferation, immunity, cardiovascular function, and neural function (Kay et al., 2016). Additionally, *DYRK1A* is associated with neurodegenerative diseases (Wegiel et al., 2011). *DYRK1A* haploinsufficiency causes *DYRK1A* syndrome (van Bon et al., 2011). Patients with *DYRK1A* syndrome often have a clinically recognizable phenotype including a typical facial gestalt, feeding problems, seizures, hypertonia, gait disturbances, and foot anomalies. Ophthalmologic, urogenital, cardiac, and/or dental anomalies have been reported. The majority of affected individuals function in the moderate-to-severe range of intellectual disability; however, individuals with mild intellectual disability have also been reported. The severity of the neurodevelopmental disorder is variable in *DYRK1A* syndrome (Courcet et al., 2012; Bronicki et al., 2015; Ji et al., 2015; van Bon et al., 2016; Evers et al., 2017; Fenster et al., 2022).

A total of 124 disease-related variants have been reported in *DYRK1A* at present (HGMD^®^ Professional 2022.2). The majority of the variants are small deletion mutations (31/124, 25.00%), missense mutations (24/124, 19.35%), nonsense mutations (21/124, 16.94%), and small insertion mutations (17/124, 13.71%) (Figure 2D, Supplementary Table S3). Although the percentage of gross deletion mutations is reported to be 10.48% (13/124), there have only been two reports of partial *DYRK1A* deletions. van Bon et al. (2011) identified a *de novo* heterozygous deletion (52 kb include exons 9–11) in *DYRK1A* using a 250K single nucleotide polymorphism array analysis. The report described a patient with intellectual disability, primary microcephaly, intrauterine growth retardation, facial dysmorphism, impaired motor functioning, and behavioral problems. Abe-Hatano et al. (2021) identified a novel 76 kb deletion containing exons 1 and 2 in *DYRK1A* using whole genome sequencing. The patient presented hypertonia, irritability, and sudden death at the age of 1 year and 7 months. The developmental quotient or IQ of these two patients was unknown, but was low.

The patient in our study with a small biparietal diameter during fetal period; birth length and weight were normal. She had normal weight development, and her height was only slightly lesser than that of peers. She presented microcephaly, binocular esotropia, bilateral valgus and external rotation, and walked with an abnormal gait. She also showed bilaterally widened lateral ventricles, developmental delay (mild motor retardation and severe language retardation), seizures, impaired social interaction, and autistic behavior. Motor, language, and hand-eye coordination; visual performance; and practical reasoning fell below the normal range. These clinical manifestations were consistent with previous reports (Courcet et al., 2012; Bronicki et al., 2015; Ji et al., 2015; van Bon et al., 2016; Evers et al., 2017). Qiao et al. identified a novel mutation c.930C>A of *DYRK1A* in a 4-year-old Chinese girl, which with typical facial dysmorphisms included deep-set eyes, pointed nasal tip, large ears, a downturned mouth, and micrognathia. From birth onward, she had feeding problems and febrile seizures, regularly. She was described as having a broadbased clumsy tread, and exhibited a mild tremor, and could not speak with full sentences and presented with lower IQ (Qiao et al., 2019). Apart from the bushy eyebrows, the patient's facial features were normal in our study, and she without feeding difficulties and gastrointestinal problems. She currently without ophthalmologic, urogenital, cardiac, or dental anomalies. In GenesReviews, van Bon et al. described the frequency of some features with *DYRK1A* syndrome were different, such as: characteristic facial features, feeding problems, gastrointestinal problems, cardiac defects, dental anomalies, and urogenital anomalies. Therefore, there are differences in the clinical phenotype of *DYRK1A* syndrome, regular follow-up is important for patients.

**Figure 3:**
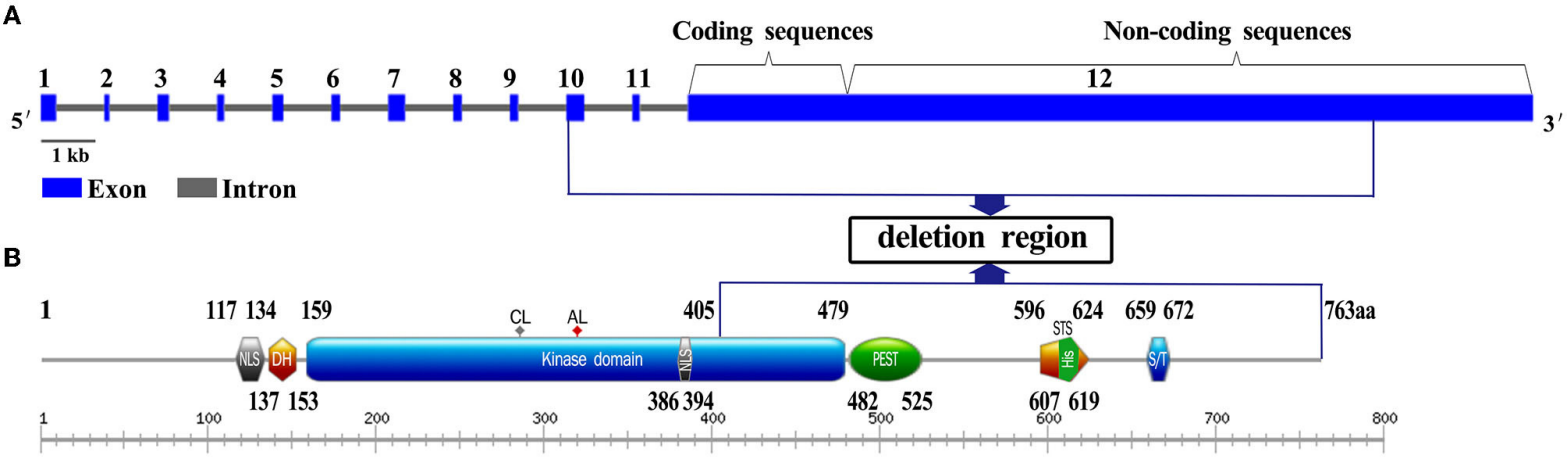
Gene and domain structure of *DYRK1A* (NM_001396.5). **(A)** The deletion region contains coding exons 10, 11, and 12, and partial sequences of non-coding exon 12 in *DYRK1A*. Introns are not in scale. **(B)** The position of the variation identified in this study is shown in the blue box. NLS, nuclear localization signal; DH, DYRK homology domain; PK, protein kinase domain; PEST, domain enriched in proline, glutamic acid, serine, and threonine residues; STS, the speckle-targeting signal; HIS, histidine repeats; S/T, serine and threonine-rich region.

DYRK1A comprises two nuclear localization signals; a DYRK homology (DH) domain; a protein kinase domain (KD); a domain enriched in proline, glutamic acid, serine, and threonine residues (PEST); a speckle-targeting signal (STS); histidine repeats (HIS); and a region rich in serines and threonines (S/T) at the C-terminus (Soundararajan et al., 2013). The 9,424 bp heterozygous deletion presented in our report contained coding exons 10, 11, and 12, and partial sequences of non-coding exon 12 in *DYRK1A* (Figure 3A). The deleted region contained a 405–763 amino acids region, which comprises the PEST, STS, HIS, and S/T repeat domains of DYRK1A (Evers et al., 2017) (Figure 3B). The mutated *DYRK1A* allele was predicted to encode a protein that lacks part of the kinase domain, which possible impairment of kinase activity. However, further research are necessary to understand the pathogenesis.

## 6. Conclusion

In this study, we described a Chinese girl who manifested microcephaly, developmental delay, seizures, autistic behavior, and impaired social interaction. Using WES, we identified a *de novo* heterozygous deletion in *DYRK1A*, which was responsible for *DYRK1A* syndrome. The *DYRK1A* variant identified in the proband was classified as pathogenic based on ACMG criteria. Although the variant was not identified in either parent, recurrent sib risks still exist because of the theoretic possibility of parental germline mosaicism. Therefore, prenatal examination of further offspring is necessary. Regular monitoring and guidance for educational and behavioral problems and growth parameters, as well as regular, lifelong follow-up is important for the proband.

## Data availability statement

The original contributions presented in the study are included in the article/Supplementary material, further inquiries can be directed to the corresponding authors.

## Ethics statement

The studies involving human participants were reviewed and approved by West China Second University Hospital of Sichuan University. Written informed consent to participate in this study was provided by the participants' legal guardian/next of kin. Written informed consent was obtained from the individual(s), and minor(s)' legal guardian/next of kin, for the publication of any potentially identifiable images or data included in this article.

## Author contributions

CZ, SL, and JW designed the study. QX, JM, and XW performed the experiments. CZ, HZ, and JW conducted data analysis. All authors read and approved the final manuscript.
