# Supplementary material for: Case report: A novel de novo deletion mutation of DYRK1A is associated with intellectual developmental disorder, autosomal dominant 7
Source: Front Neurosci. 2023 May 19;17:1174925. doi: 10.3389/fnins.2023.1174925 (PMC10235440; doi:10.3389/fnins.2023.1174925)
Supplement: Supplementary file 1 [file Data_Sheet_1.docx]

Supplementary Material

Case report: A novel de novo deletion mutation of *DYRK1A* is associated with intellectual developmental disorder, autosomal dominant 7

Cong Zhou^1 2^, Hongmei Zhu^1 2^, Qinqin Xiang^1 2^, Jingqun Mai^1 2^, Xihan Wang^1 2^, Jing Wang^1 2*†^, Shanling Liu^1 2*†^

*** Correspondence:** †These authors have contributed equally to this work and share Corresponding author

^1^ Department of Medical Genetics, Prenatal Diagnostic Center, West China Second University Hospital, Sichuan University, Chengdu, China

^2^ Key Laboratory of Birth Defects and Related Diseases of Women and Children, Ministry of Education, Sichuan University, Chengdu, China

20 Section 3 Renmin South Road, chengdu, Sichuan 610041, P.R. china

E-mail (Jing Wang): hhwj_123@163.com

E-mail (Shanling Liu): sunny630@126.com

# Supplementary Tables

Table S1. qPCR primers for exon 9, 10 and 12 of the *DYRK1A.*

| Exon | Primers | Sequence | Size(bp) | Genomic location |
| --- | --- | --- | --- | --- |
| exon 9 | exon 9-F | TAGTGGAAGTTCTGGGTAT | 97 | chr21:37496137_37496233 |
| exon 9 | exon 9-R | AAGTTCCAAGTGCCATCT |  |  |
| exon10 | exon10-F | ATACGGTCGCTGACTACT | 182 | chr21:37505374_37505555 |
| exon10 | exon10-R | AGACTGAGACTGCTCCAT |  |  |
| exon12 | exon12-5’-F | TTCCTGCTCCTCTTGGTT | 152 | chr21:37511924_37512075 |
| exon12 | exon12-5’-R | GTGGTGGTGATGGTGATG |  |  |
| exon12 | exon12-3’-F | CTGCTAAGATGTGTTATGACTG | 198 | [chr21:37525147_37525344](http://genome.ucsc.edu/cgi-bin/hgTracks?hgsid=1610213417_PRf4sRYvE7KcPA7ARzVT4oKOqvUA&db=hg38&position=chr21:37525147-37525344&hgPcrResult=pack) |
| exon12 | exon12-3’-R | CACCTAAGACTTGCCTTGG |  |  |

Table S2. Summary of whole exome sequencing on the patient's family members.

| Statistics data indicators | Statistical results | | |
| --- | --- | --- | --- |
|  | Proband | Mother | Father |
| Clean bases(Mb) | 16573.82 | 21547.43 | 16931.37 |
| Clean reads,n | 113336616 | 147368994 | 115988752 |
| Mapped reads,n | 104497180 | 135662653 | 106793723 |
| Mapped,% | 92.20% | 92.06% | 92.07% |
| Unique reads,n | 70195531 | 90212071 | 74330604 |
| Unique,% | 67.17% | 66.50% | 69.60% |
| Reads uniquely mapped to genome,n | 70195531 | 90212071 | 74330604 |
| Reads uniquely mapped to target,n | 55538599 | 72049893 | 57220170 |
| Fraction of uniquely mapped on target,% | 79.12% | 79.87% | 76.98% |
| Total effective yield(Mb) | 10172.32 | 13058.78 | 10740.49 |
| Total sequences on target(Mb) | 6174.08 | 8036.02 | 6329.22 |
| Fraction of effective bases on target,% | 60.69% | 61.54% | 58.93% |
| Total bases on target,n | 56659996 | 56659996 | 56659996 |
| Base covered on target,n | 56468649 | 56480161 | 56532341 |
| Coverage of target region,% | 99.66% | 99.68% | 99.77% |
| Average sequencing depth on target(X) | 108.97 | 141.83 | 111.71 |
| Fraction of target covered at least 20X,% | 99.15% | 99.28% | 99.28% |

Table S3. Disease mutation of *DYRK1A* gene (NM_001396.5) according to The Human Gene Mutation database (Professional 2022.2).

| Mutation type | Number of mutation | Ratio |
| --- | --- | --- |
| Nonsense | 21 | 16.94% |
| Missense | 24 | 19.35% |
| Splicing | 9 | 7.26% |
| Small deletions | 31 | 25.00% |
| Small insertions | 17 | 13.71% |
| Small indels | 3 | 2.42% |
| Gross deletions | 13 | 10.48% |
| Gross insertions | 3 | 2.42% |
| Complex | 3 | 2.42% |
| Total | 124 | 100 |
